# Harnessing non-covalent interactions to exert control over regioselectivity and site-selectivity in catalytic reactions

**DOI:** 10.1039/c6sc04157d

**Published:** 2016-10-05

**Authors:** Holly J. Davis, Robert J. Phipps

**Affiliations:** a Department of Chemistry , University of Cambridge , Lensfield Road , Cambridge , CB2 1EW , UK . Email: rjp71@cam.ac.uk

## Abstract

Asymmetric catalysis has been revolutionised by the realisation that attractive non-covalent interactions such as hydrogen bonds and ion pairs can act as powerful controllers of enantioselectivity when incorporated into appropriate small molecule chiral scaffolds. Given these tremendous advances it is surprising that there are still a relatively limited number of examples of non-covalent interactions being harnessed for control of regioselectivity or site-selectivity in catalysis, two other fundamental selectivity aspects facing the synthetic chemist. This perspective examines the progress that has been made in this area thus far using non-covalent interactions in conjunction with transition metal catalysis as well as in the context of purely organic catalysts. We hope this will highlight the great potential in this approach for designing selective catalytic reactions.

## Abstract 2

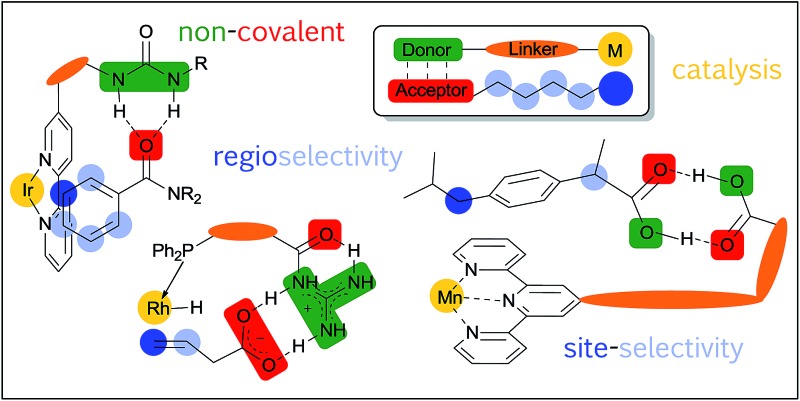
This perspective examines the progress that has been made in using non-covalent interactions to control regioselectivity and site-selectivity in catalysis.

## 1 Introduction

The past fifteen years have seen remarkable advances in the use of non-covalent interactions as key activating and controlling elements in the field of asymmetric catalysis.^1^ The area of non-covalent asymmetric organocatalysis is now arguably developing at a faster rate than the covalent variety as new arenas are explored for modes of catalysis which primarily, but not exclusively, employ hydrogen bonding^2–4^ and ion pairing^5–8^ interactions. This recent direction is in somewhat sharp contrast to earlier developments in asymmetric catalysis, which almost solely relied on dative covalent substrate–metal–ligand interactions.^9^ Whilst non-covalent interactions had been implicated in some key processes (*e.g.* Noyori transfer hydrogenation^10^ and oxazaborolidine-catalysed reactions^11^), they were generally regarded as of secondary importance. This contrasts with enzymatic catalysis, where non-covalent interactions are crucial to the mechanism of catalysis.^12^ Perhaps the forbidding complexity of enzymes gave the impression that attempting to use these same interactions in the context of small molecule catalysts would be futile? Whilst pioneering early work towards ‘artificial enzymes’ sought to replicate to some degree the macromolecular structures involved,^13,14^ the relatively recent developments in non-covalent organocatalysis have demonstrated that multiple non-covalent interactions orchestrated by small molecule chiral catalysts can be tremendously powerful tools for controlling enantioselectivity.^1–3,5–7^ Despite these advances, one should not forget that enantioselectivity is not the only important aspect of selectivity to be controlled during chemical synthesis. Often just as crucial, if not more so, are chemoselectivity and regioselectivity, as articulated by Bode and co-workers in their insightful 2012 review on selective catalysis.^15^ Indeed, in enantioselective catalysis at least the playing field is level, there being no innate selectivity for one enantiomer over the other. In a regioselectivity context, for example, it is quite possible that natural selectivity may need to be overridden to access a particular isomer.

In this perspective we aim to examine progress made thus far in using non-covalent interactions to control regioselectivity and site-selectivity in catalysis. For the purposes of this article, we have opted to define regioselectivity as being a choice between different positions within what can be reasonably defined as a single functional group (Section 2).^15,17^ When there is more than one instance of a particular functional group, such as a C–H bond, present in a molecule and these are not located within the same functional group, we will refer to this situation as one of site-selectivity, a specific type of chemoselectivity (Section 3).^18,19^ In order to provide focus, we will not discuss the control of chemoselectivity that may occur between two formally different functional groups within a molecule (such as an alkene and an alkyne), although we acknowledge that this distinction can become blurred when groups that formally belong to the same class bear quite different substitution. Finally, in order to emphasise the latest advances in this emerging field, which we believe will be of most interest to the reader, we will focus on developments in catalytic reactions. As such we will not cover use of non-covalent interactions for direction of reagents in reactions that do not involve a catalyst, such as hydrogen bond-directed epoxidation with peracids, although such strategies are no doubt very powerful and have been previously discussed elsewhere.^20,21^ Having said this, we will touch upon several examples which are not rendered catalytic, but which we think offer insights relevant to the discussion herein and which could potentially be amenable to catalysis. There are a number of previous articles which also address elements that will be covered herein, including those which focus on transition metals^22,23^ and those which approach from a viewpoint of supramolecular catalysis.^14,24–26^ Due to the emerging nature of this area as defined above, there are not an excessive number of examples and as such we believe that we have been able to cover the majority of these herein. In some cases, the proposed mechanism and origin of selectivity may be more speculative than in others where more detailed studies have been undertaken, but where possible we have provided the rationale given in the original publication in an attempt to provide maximal insight into the possible role played by the non-covalent interaction. Given the remarkable impact that the employment of non-covalent interactions has had on recent developments in enantioselective catalysis, together with their crucial role in enzymatic catalysis, we believe that the potential for synthetic chemists to apply them to control regioselectivity and site-selectivity aspects is ample. This potential is particularly pertinent due to the rapid recent progress in C–H bond functionalisation chemistry where one of the key challenges faced is that of obtaining selectivity in the presence of multiple potentially reactive C–H bonds in a molecule.^16^ There are also ample opportunities for judicious application of non-covalent interactions to control selectivity in well-established reactions, for example as seen later in this article in the context of hydroformylation. We hope that this inspires further developments in this emerging field.

## 2 Control of regioselectivity in catalytic reactions using non-covalent interactions

As mentioned in the introduction, we have opted to define regioselectivity as being a choice between different positions within what can be reasonably defined as a single functional group. The section will be subdivided according the functional group undergoing reaction, with the majority of transformations in this class concerning arenes (Section 2.1), alkynes (Section 2.2) and alkenes (Section 2.3).^15,17^ The predominant way in which non-covalent interactions have been employed with these functionalities has been in conjunction with a reactive transition metal. Generally, a customised ligand for the metal also possesses a site with which to interact with the substrate *via* one or two hydrogen bonds or, less commonly, an ion pair. In Section 2.4, a single example of regioselective ketone functionalisation is given and in this case an organic peptide catalyst is employed, a scenario that will be explored in more detail in Section 3.

### 2.1 Regioselective functionalisation of arenes

**Fig. 1:**
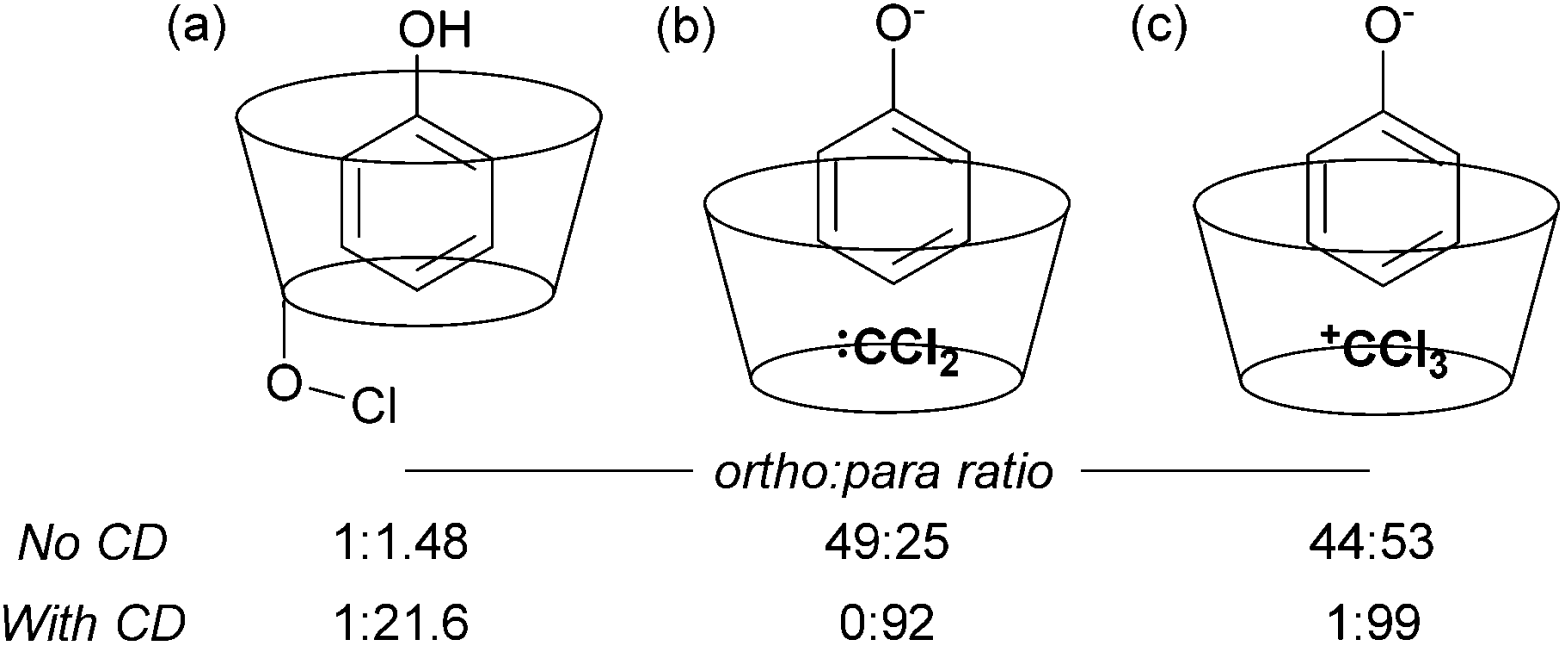
(a) Proposed mode of reaction for *para*-chlorination of anisole bound inside α-cyclodextrin (CD). (b) Rationalisation of selectivity in *para*-selective formylation of phenol catalysed by β-cyclodextrin. (c) The same for carboxylation.

The issue of control of regioselectivity in arene functionalisation is perhaps one of the best-articulated, introduced from an early level of chemical education. Limitations in this area are an obvious target to address using non-covalent interactions, a notable one being that the majority of directed approaches catalysed by transition metals result in proximal isomers due to the necessity for the substrate to interact directly with the metal centre. Since the 1960s, cyclodextrins have attracted great interest due to the opportunities provided by the hydrophobic cavity to act as a microvessel for chemical reactions by virtue of the hydrophobic effect, with potential implications for rate and selectivity.^27^ Breslow and Campbell carried out pioneering work on controlling the selectivity of anisole chlorination through binding within a cyclodextrin, albeit this was not used in catalytic amounts.^28^ Not only was the chlorination highly selective but the rate was increased which led the authors to hypothesise catalysis *via* a cyclodextrin hydroxyl being converted into a hypochlorite which then acts as the true donor (Fig. 1a). Subsequently, cyclodextrins were demonstrated to be usable in catalytic amounts for *para*-selective formylation^29^ and carboxylation^30^ amongst other reactions.^31^ In these specific cases, studies showed that in the presence of catalytic cyclodextrin, excellent *para* selectivity was obtained when otherwise poor *ortho*/*para* selectivity resulted (Fig. 1). Mechanistic studies suggested that dichlorocarbene (Fig. 1b, for formylation) or the trichloromethyl cation (Fig. 1c, for carboxylation) occupied the cyclodextrin cavity and that the arene substrate penetrated only shallowly to avoid inclusion of the polar phenoxide group, resulting in close proximity of the *para* carbon to the reactive species.

**Fig. 2:**
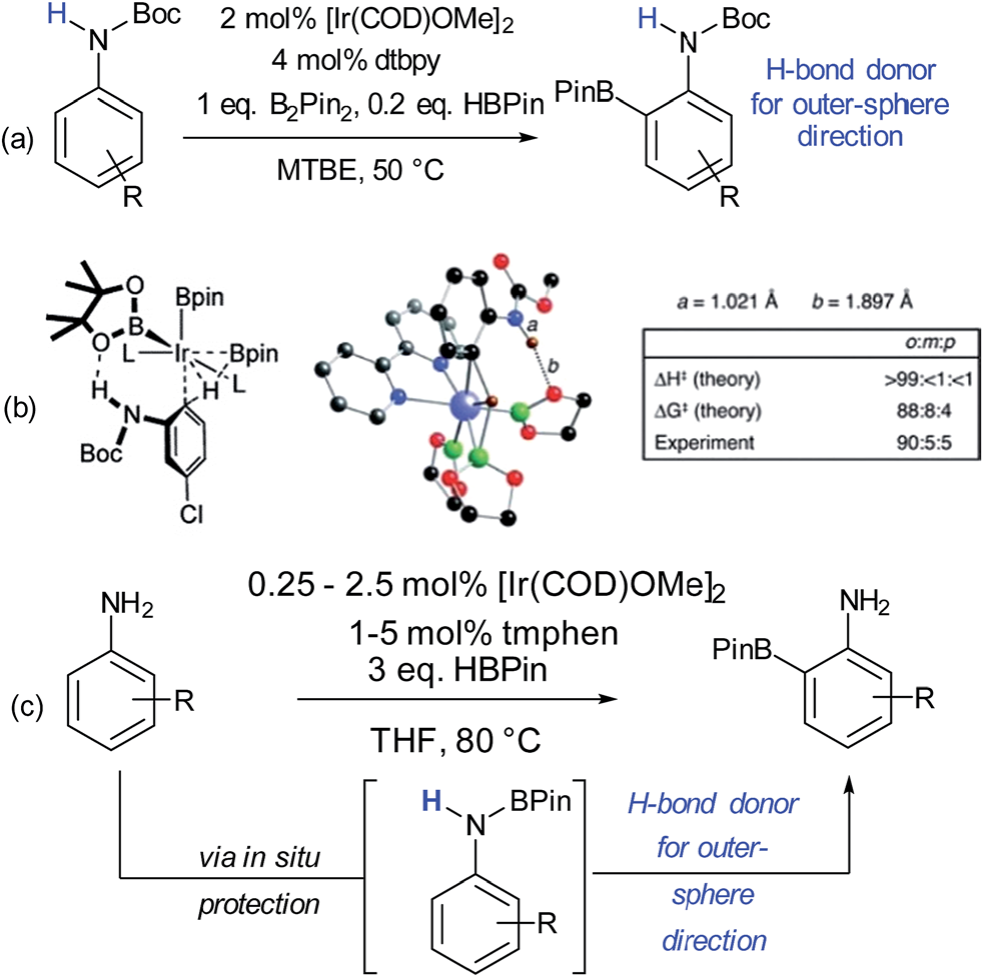
(a) *ortho*-Selective borylation of *N*-Boc anilines. (b) Schematic of proposed transition state for reaction together with results of theoretical predictions and calculations of N–H and O–H bond distances in the TS. (c) *ortho*-Selective borylation of anilines by *in situ* formation of *N*-BPin derivative. (b) Reprinted with permission from ref. 35. Copyright 2012 American Chemical Society.

Since these early advances, few explicit examples of regiocontrol in catalytic arene functionalisation using non-covalent interactions occurred until relatively recently. The iridium-catalysed borylation of aromatic C–H bonds is a method notable among other C–H activation methods for its mild conditions, effectiveness in non-polar solvents and lack of requirement for acidic additives.^32^ All of these factors make it an attractive platform on which to investigate applying non-covalent interactions, which may be easily disrupted by more aggressive reaction conditions. Selectivity in iridium-catalysed borylation is well established to be primarily based on steric factors in the absence of directing groups, with mono-substituted and 1,2-disubstituted arenes generally resulting in regioisomeric mixtures.^33,34^ In 2012, Singleton, Maleczka, Smith and co-workers made the unusual observation that on 3-substituted *N*-Boc anilines, borylation occurs both at the *ortho* and *meta* positions, rather than just the sterically preferred *meta*-position (Fig. 2a).^35^ Mechanistic studies suggested that the selectivity could be attributed to a hydrogen bond between the substrate N–H and an oxygen acceptor on one of the pinacol ligands of the active iridium catalyst complex, which the authors termed ‘outer-sphere direction’, with theoretical calculations supporting the substrate–ligand hydrogen bond hypothesis (Fig. 2b). The requirement for the carbamate group was quite specific – simply changing to an amide resulted in standard sterically-dictated selectivity. Also, high selectivity could only be achieved with anilines bearing a *para*-substituent, thereby limiting the scope, as possibly the pinacol oxygen is a relatively weak H-bond acceptor and in some cases steric priorities override the directing effect. The same group subsequently reported that whilst free anilines are poor substrates for borylation, they can be readily *N*-borylated *in situ* by treatment with excess pinacol borane under the standard borylation conditions (Fig. 2c).^36^ These *N*-boryl anilines are likely to also be competent hydrogen bond donors for directing borylation to the *ortho* position according to the previously proposed mechanism.

**Fig. 3:**
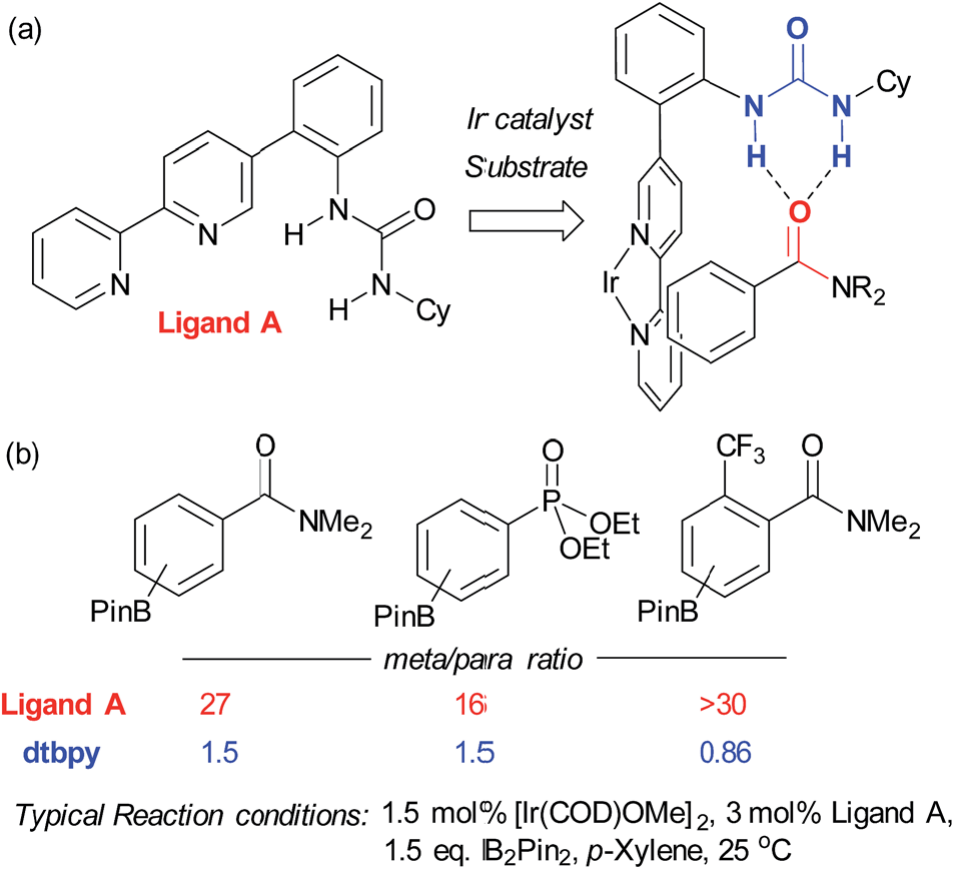
(a) The optimal bifunctional bipyridine ligand bearing a hydrogen bond-donor urea, together with the catalyst–substrate complex thought responsible for *meta*-selective borylation. (b) A selection of substrates, highlighting *meta*:*para* selectivity for both the optimal bifunctional ligand and standard borylation ligand dtbpy.

In contrast to this approach, wherein the substrate interacts with functionality already present in the standard catalyst, Kuninobu, Kanai and co-workers recently reported a novel ligand-directed borylation strategy which enabled *meta*-selective borylation on a selection of mono-substituted and 1,2-disubstituted arenes.^37^ Their strategy is based on incorporation of a hydrogen bond donor into the bipyridine ligand scaffold in a position such that it would be able to interact with a hydrogen bond accepting carbonyl group on the substrate (Fig. 3a). They synthesised several catalyst analogues with the urea group in different positions, demonstrating that an *ortho*-substituted arene in the bipyridine 5-position gives the highest *meta* selectivity, presumably through the optimal spatial arrangement of the complexed catalyst and substrate in the transition state. They show that the carbonyls of both amides and phosphonates are competent hydrogen bond acceptors, leading to very high *meta*-selectivity for a number of substrates (Fig. 3b) and carried out a series of studies with methylated catalysts, which provided strong evidence for their hydrogen bond-directed hypothesis for regiocontrol.

**Fig. 4:**
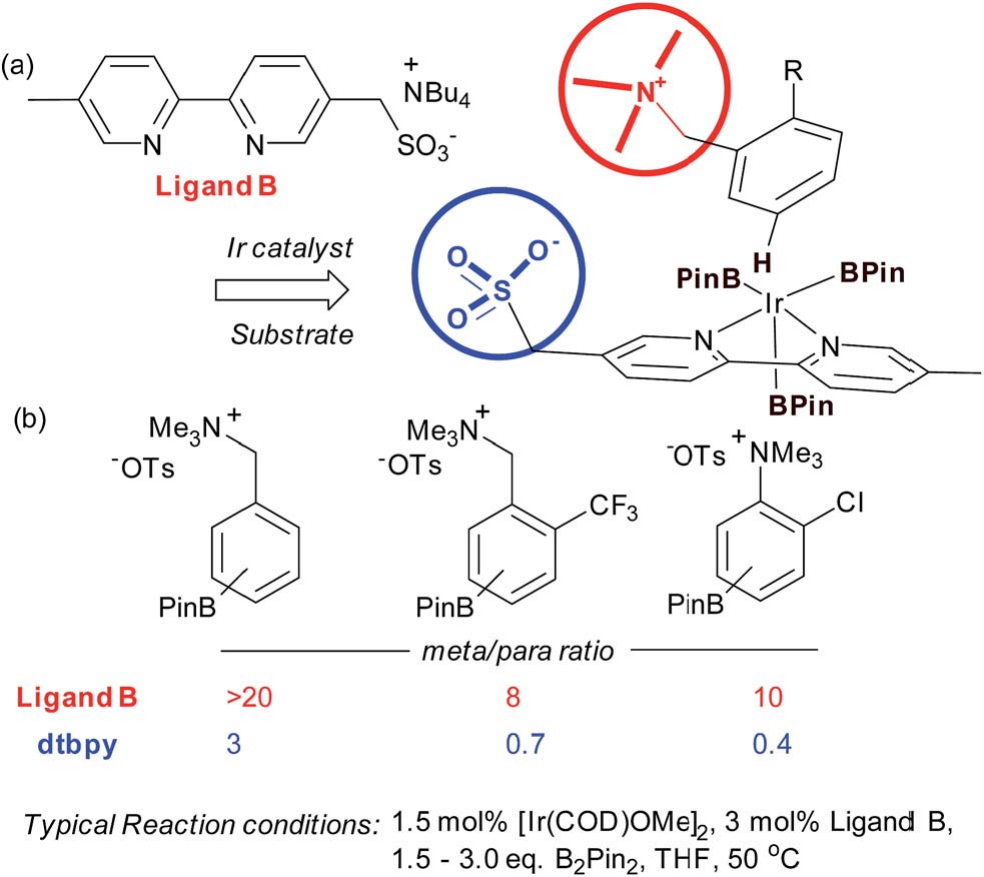
(a) Optimal ligand design bearing anionic sulfonate group together with working hypothesis for ion-pair directed borylation. (b) Selection of substrates, highlighting *meta*:*para* selectivity for both the anionic ligand B and dtbpy.

Very recently, our group has reported an approach which utilises an ion-pairing interaction between catalyst and substrate to achieve *meta*-selective borylation of aromatic quaternary ammonium salts.^38^ This represents a rare case of an ion-pairing interaction being employed to direct transition metal catalysis to achieve regiocontrol; previous advances have focussed solely on enantiocontrol.^5–7,39,40^ A series of bipyridines bearing an anionic sulfonate group in different positions were investigated and the optimal configuration was found to include a methylenesulfonate group in the 5-position of the bipyridine (Fig. 4a, ligand B). This was effective for the *meta*-selective borylation of two classes of aromatic quaternary ammonium salts, those derived from anilines and benzylamines. These cationic substrates were envisaged to participate in an ion exchange with the iridium-bound ligand to form the reactive complex, able to direct borylation to the arene *meta* position (Fig. 4a). A selection of substrates is shown in Fig. 4b, showing the greatly improved selectivity using the anionic ligand B compared with standard ligand dtbpy.

**Fig. 5:**
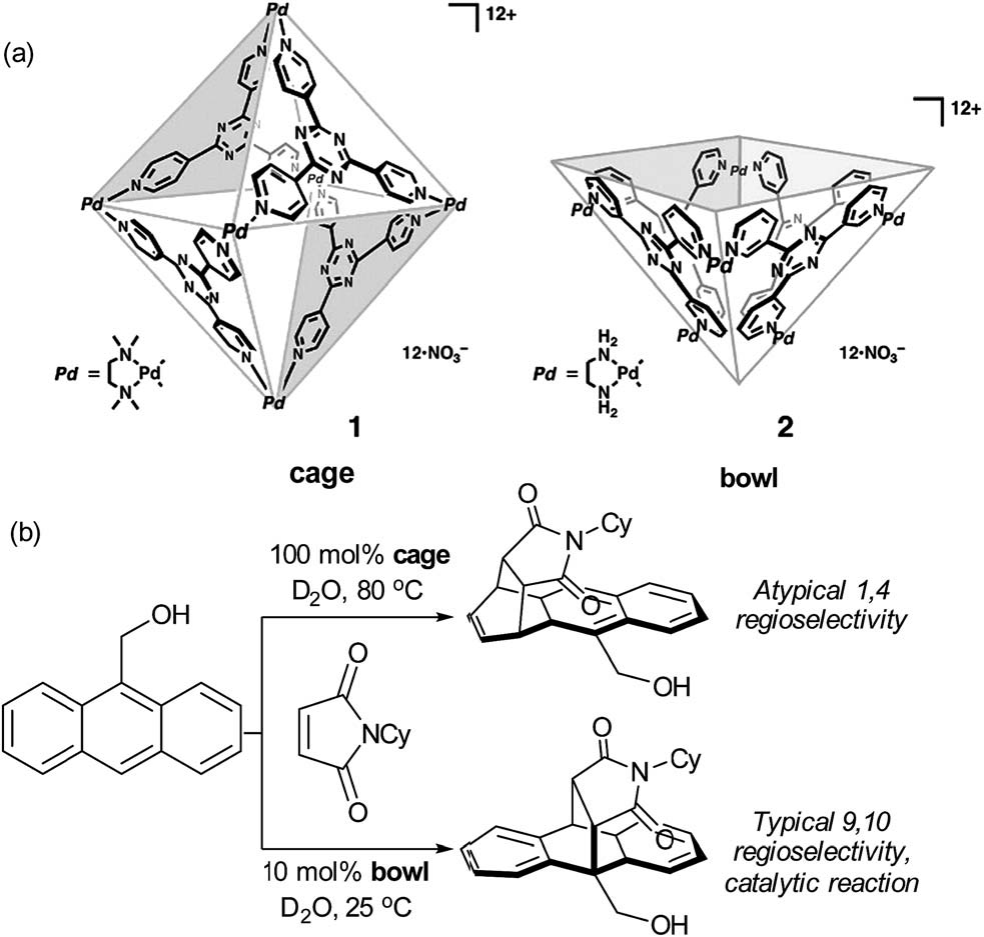
(a) An octahedral cage and square-pyramidal bowl assembled from *cis* end-capped Pd(ii) ions and triazine-cored tridentate ligands. (b) Divergent regioselectivity obtained in the presence of the cage and bowl structures in the Diels–Alder reaction of anthracenes with phthalimides. (a) Reprinted with permission from ref. 41. Copyright 2006 The American Association for the Advancement of Science.

Though not a catalytic reaction, the regioselectivity switch observed by Fujita and co-workers when carrying out the Diels–Alder reaction between anthracene and phthalimide guests within an aqueous organopalladium cage highlights the remarkable potential of these self-assembled hosts for accessing unconventional regioselectivity.^26^ Specifically, a confined cage and a related open-topped bowl gave opposite regioselectivity, with the cage giving atypical 1,4-regioselectivity for addition across the naphthalene ring (Fig. 5a and b).^41^ This was attributed to the limited space in the hydrophobic cage forcing the dienophile double bond to be proximal to the 1,4 position of the anthracene; the orientation required for the electronically favoured 9,10 position to react is likely to be unobtainable. Favourable product binding unfortunately precludes turnover in this case. The less restricted bowl, which allows the 9,10 regioisomer formation, is able to function as a catalyst since the ‘bent’ geometry of the product disfavours aromatic stacking interactions compared with the starting material. Related studies on regioselective naphthalene Diels–Alder reactions were later disclosed.^42^

### 2.2 Regioselective functionalisation of alkynes

**Fig. 6:**
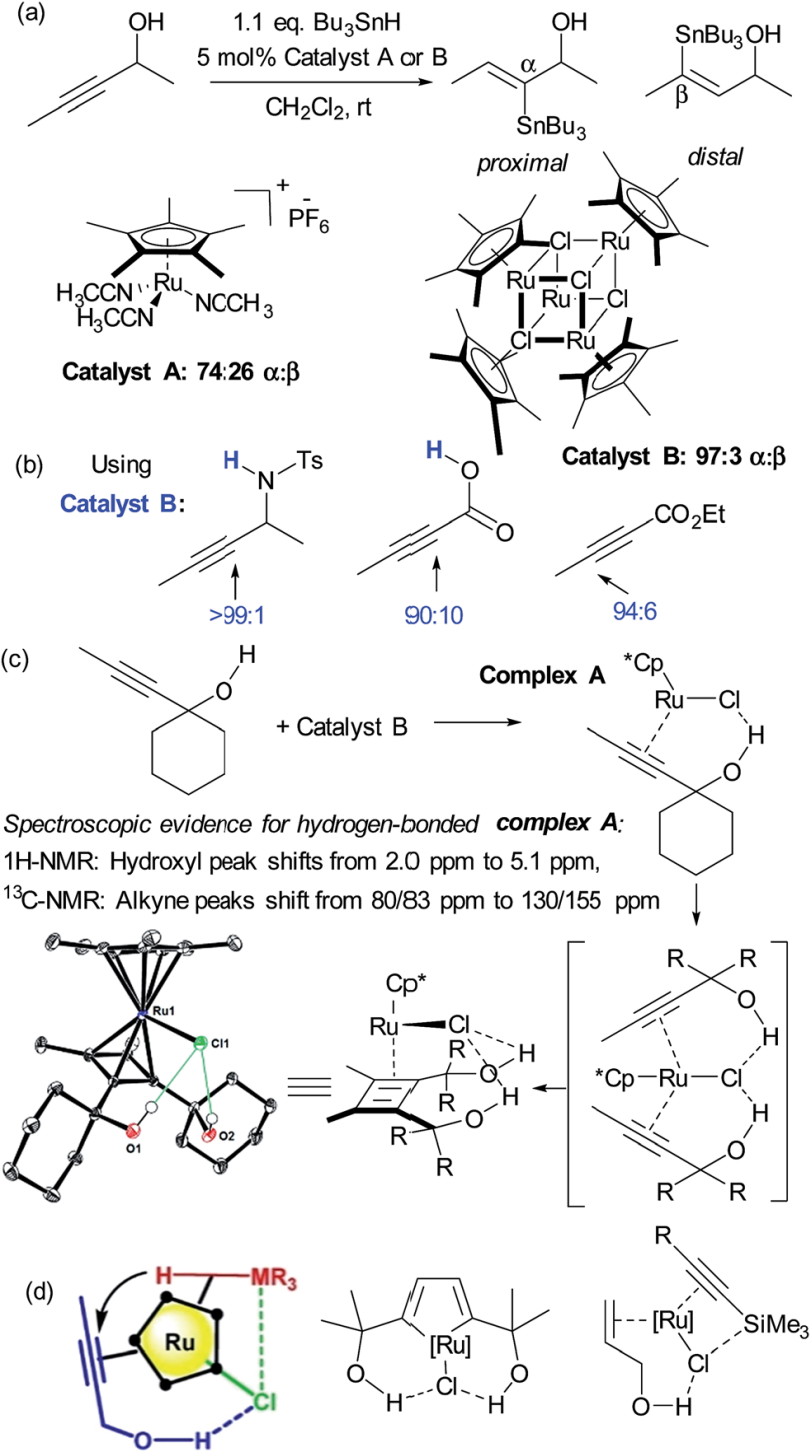
(a) *trans*-Selective hydrostannation of propargylic alkynes using ruthenium chloride complex B as opposed to cationic variant A. (b) High regioselectivity is also observed in other unsymmetrical alkynes bearing protic functionality, with selectivity reversal in the case of an ester. (c) Selected experiments conducted to probe the role of hydrogen bonding in the observed regioselectivity. (d) Hypothesis for origin of regioselectivity (left), plus application of this hypothesis to explain observed regioselectivity in two previously reported ruthenium catalysed reactions. (c) and (d) Reprinted with permission from ref. 45. Copyright 2015 American Chemical Society.

Hydrometalation reactions of unsymmetrical alkynes are often challenging to control in terms of regioselectivity and hydrogen bond directed catalysis has been demonstrated to be powerful to this end.^43^ In 2014, Fürstner and Rummelt reported the stereoselective *trans*-hydrostannation of alkynes catalysed by cationic ruthenium Cp* complexes.^44^ In this report they made the interesting observation that unsymmetrical alkynes bearing propargylic alcohols delivered poor regioselectivity with a cationic ruthenium complex (Fig. 6a, catalyst A) but excellent regioselectivity for the proximal position when using a related non-cationic ruthenium chloride complex (catalyst B). Excellent proximal selectivity was also observed using the ruthenium chloride complex on a propargylic sulfonamide and carboxylic acid, both bearing protic functionality. Furthermore, an analogous ester which bears no protic functionality gave opposite, distal selectivity (Fig. 6b). Fürstner and co-workers subsequently disclosed that this also applies to *trans*-hydrosilylation and hydrogermylation and undertook a very detailed mechanistic investigation in an attempt to shed light on its origin.^45^ This consisted of NMR studies which strongly suggested metal-substrate complexation occurring through hydrogen bonding when catalyst B was employed, but crucially not cationic catalyst A (Fig. 6c). Also, X-ray structures of several related complexes were obtained after ligand dimerisation, which show clear interligand hydrogen bonds between the chloride ligand on Ru and the dimer hydroxyl groups (Fig. 6c). Their conclusion from these and other experiments was that the [Ru–Cl] unit of the catalyst may serve as a hydrogen bond acceptor, leading to a highly favourable organisation of the catalyst with bound propargylic alkyne through an interligand hydrogen bond. A further interaction between the chloride and the heteroatom of the stannane, silane or germane was hypothesised, based on further experiments, to potentially reinforce this arrangement and the authors suggested that a highly preorganised complex could explain the enhanced regioselectivity and reaction rates (Fig. 6d, left). The authors used their hypothesis to shed light on hitherto unexplained regioselectivity in two further ruthenium catalysed reactions in which Ru–Cl functioning as a hydrogen bond acceptor can now provide a very satisfactory rationalisation (Fig. 6d, centre^46^ and right^47^). Another interesting example of alkyne functionalisation is from Grotjahn and co-workers, who have developed a ruthenium catalyst able to perform the anti-Markovnikov hydration of alkynes with very high selectivity and rates.^48^ The key was the use of phosphine ligands bearing basic heteroatoms which are thought to engage in hydrogen bonding and proton transfer to stablise numerous transition states along the proposed reaction pathway.^49,50^

### 2.3 Regioselective functionalisation of alkenes

**Fig. 7:**
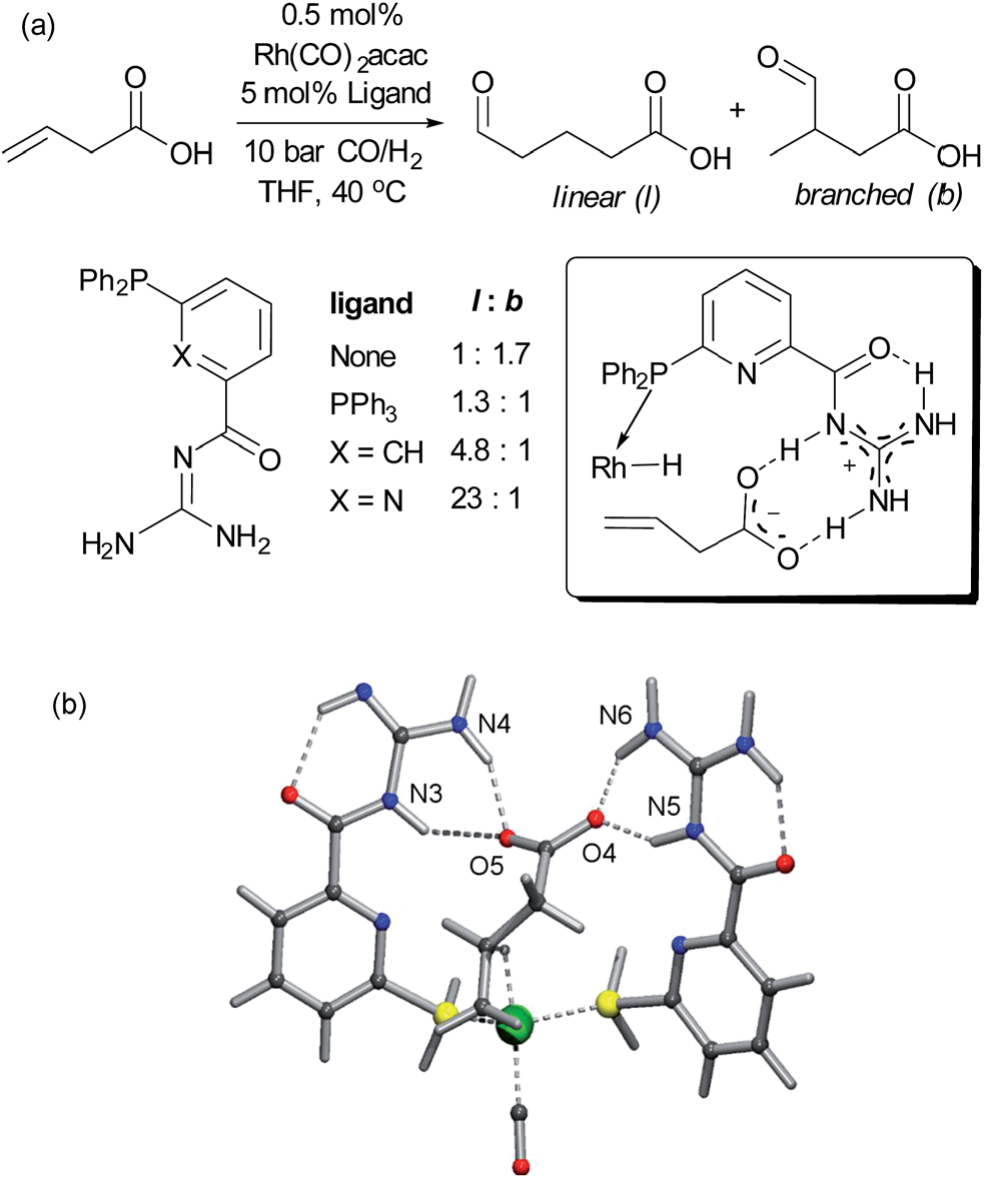
(a) Evaluation of ligands for the hydroformylation of β,γ-unsaturated carboxylic acids and original hypothesis for control of regioselectivity. (b) Calculated transition state for reaction which involves interactions between two bifunctional ligands and the substrate. (b) Reprinted with permission from ref. 53. Copyright 2010 Wiley.

Alkenes are versatile functional groups in which there can often be a regioselectivity choice upon elaboration. Their numerous reactions with transition metals mean control of regioselectivity through non-covalent interactions could very be an attractive strategy with an appropriate multifunctional ligand. Advances to this end have so far focussed on regioselective hydroformylation of alkenes. In 2008, Šmejkal and Breit reported an acylguanidinium-functionalised phosphine ligand for rhodium wherein the guanidine portion engages in a dual hydrogen bonding interaction with the carboxylic acid functionality of a suitable alkene substrate (Fig. 7a).^51^ It was intended through catalyst design to influence the regioselectivity of the reaction, which gave poor selectivity using no ligand, or triphenylphosphine. The optimal catalyst not only gave >20 : 1 linear : branched (l : b) selectivity but also greatly increased turnover number. The hypothesised substrate binding is shown (Fig. 7, inset) and support for this hypothesis is provided by the fact that the corresponding methyl ester gives poor selectivity. Additionally, selectivity drops off as the alkene is moved further away from the carboxylic acid, decreasing to 3.6 : 1 l : b on insertion of an extra carbon, which presumably extends beyond the ‘reach’ of the metal centre. Furthermore, addition of extraneous carboxylic acids reduced selectivity. The optimal catalyst was also shown to be very effective for challenging internal alkene substrates, which are generally not only less reactive but possess very similar electronic properties at both carbon atoms. This success provided further support for the directed nature of the hydroformylation. The same authors subsequently developed a directed hydroformylation/decarboxylation process^52^ and reported a complete study in which more active catalysts were developed and the mechanism probed computationally.^53^ Interestingly, the calculations suggested that the lowest energy transition state that arises involves two bifunctional ligands on the metal interacting with the carboxylate of the substrate, in contrast to the original hypothesis (Fig. 7b).

**Fig. 8:**
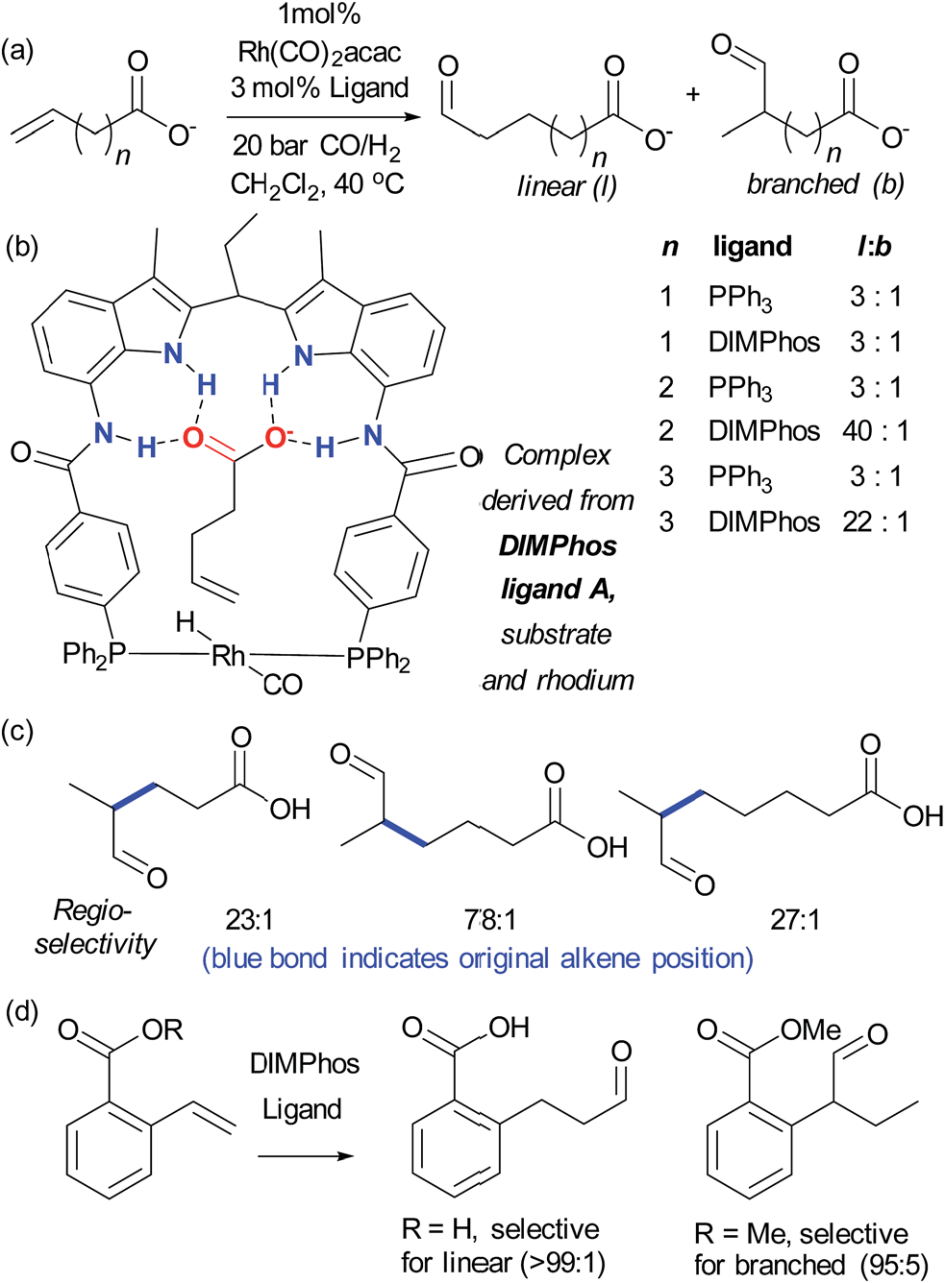
(a) Highly regioselective hydroformylation of a range of unsaturated alkene chain lengths, except for *n* = 1, using ligand A compared to using PPh_3_. (b) Active complex for hydroformylation as determined by mechanistic studies and DFT calculations. (c) Illustration of high regioselectivities achievable on internal alkenes of variable chain lengths. (d) A phosphite-based DIMPhos ligand is able to invert the standard regioselectivity for hydroformylation of 2-carboxylvinylarenes.

Reek and co-workers tackled the regioselective hydroformylation of alkenes by installing a rhodium binding site into an anion receptor scaffold with the aim of precisely controlling the geometry of the transition state through multiple substrate–catalyst hydrogen bonding interactions (Fig. 8b).^54^ They found that the highest l : b selectivity was observed for the 4-pentenoate anion, as this is thought to exactly span the distance between the metal centre and the receptor portion. Excellent selectivity was also seen with longer acids and, in agreement with calculations, 3-butanoate was too short to reach the receptor and thus gave poor results with the original catalyst (Fig. 8a). The carboxylate functionality was found to be crucial – ester or neutral carboxylic acid analogues gave poor selectivity, concurring with the anion-binding hypothesis, and an unsaturated phosphate was also found to undergo highly selective hydroformylation. They subsequently examined challenging internal alkenes.^55^ These substrates required modification of the catalyst to incorporate more reactive phosphite ligands, and the resulting catalyst was highly selective for a range of branched substrates. In all cases the aldehyde was delivered to the more distant alkene carbon, a very impressive example of remote regiocontrol (Fig. 8c). Detailed mechanistic studies were carried out and calculations suggested that the nature of the anion binding restricts alkene rotation alkene, lowering the energy of the pathway leading towards the observed products.

The same authors have also applied their approach to the selective hydroformylation of vinyl arenes bearing a carboxylic acid group, in order to access β-aryl aldehydes.^56^ Calculations suggested that the catalyst should favour the linear product through stabilisation of the transition state leading to the β-phenylalkyl rhodium complex and indeed excellent regioselectivity was observed whilst the analogous ester resulted in predominantly the branched regioisomer, presumably through its inability to bind to the catalyst (Fig. 8d). This transformation was subjected to detailed mechanistic studies and also demonstrated to be highly effective on substrates bearing an internal double bond, on allyl arenes and to some extent *meta*-carboxyl vinyl arenes although in the latter case selectivity was lower presumably due to a poorer ‘fit’ in the binding pocket.^57^

### 2.4 Regioselective functionalisation of ketones

**Fig. 9:**
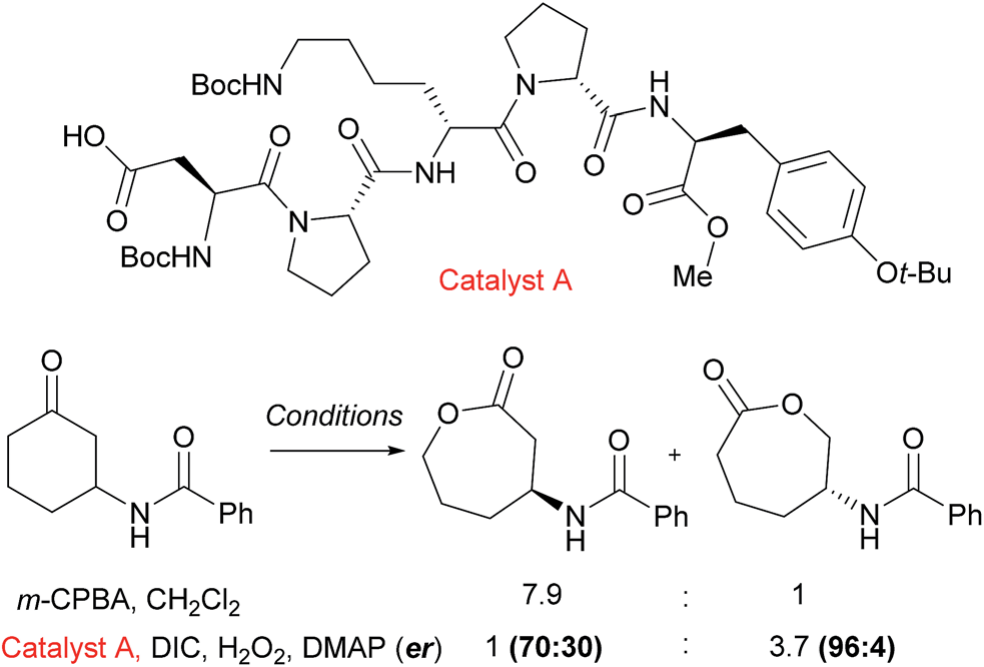
Reversal of regioselectivity observed in Baeyer–Villiger oxidation of particular cyclic ketones using catalyst A when compared with *m*CPBA.

An example from the Miller group using their considerable expertise with peptide catalysts^58^ demonstrates catalyst control in the regioselectivity of Baeyer–Villiger oxidation of cyclic ketones bearing amide functionality.^59^ Mechanistically, the catalysis occurs by the carboxylic side chain of a terminal residue being activated with a carbodiimide (DIC) and then attacked by hydrogen peroxide to form a transient peroxyacid at the peptide terminus. The selectivity was hypothesised to be a result of hydrogen bonding interactions between the substrate N–H and an acceptor in the peptide, as the presence of the hydrogen bond donor was important to obtain catalyst control in the reaction. It was noted from an initial screen that the presence of a “Pro-DXaa-Pro” sequence was conserved between multiple hits and this motif was explored and elaborated to ultimately yield peptide catalyst A (Fig. 9). As well as offering control of regioselectivity, catalyst A also was also capable of displaying high levels of enantioselectivity as well as enrichment of the starting material in a parallel kinetic resolution process, which worked most effectively when a urea unit was incorporated into the substrate (not shown). This approach to peptide peracid catalysis has recently be expanded by the group where they are now able to control regio-, chemo- and diastereoselectivity through use of carefully chosen peptide catalysts.^60^

## 3 Control of site-selectivity in catalytic reactions using non-covalent interactions

As mentioned in the introduction, when there is more than one instance of a particular functional group, such as a C–H bond, present in a molecule and these are not located within the same functional group, we will refer to this situation as one of site-selectivity, a specific type of chemoselectivity.^18,19^ Again, this section will be subdivided according the nature of functionality undergoing reaction. As in the majority of Section 2, approaches to site-selective aliphatic C–H functionalisation (Section 3.1) see the non-covalent apparatus paired with a reactive transition metal. In contrast, in the bulk of the subsequent sections covering alkenes (Section 3.2) and particularly polyols (Section 3.3), it is organic catalysts that are most effective. These often possess an extended peptide backbone which offers numerous opportunities for engaging in non-covalent interactions with ‘matched’ substrates, in a manner very much akin to the way that enzymes function.

### 3.1 Site-selective aliphatic C–H functionalisation

**Fig. 10:**
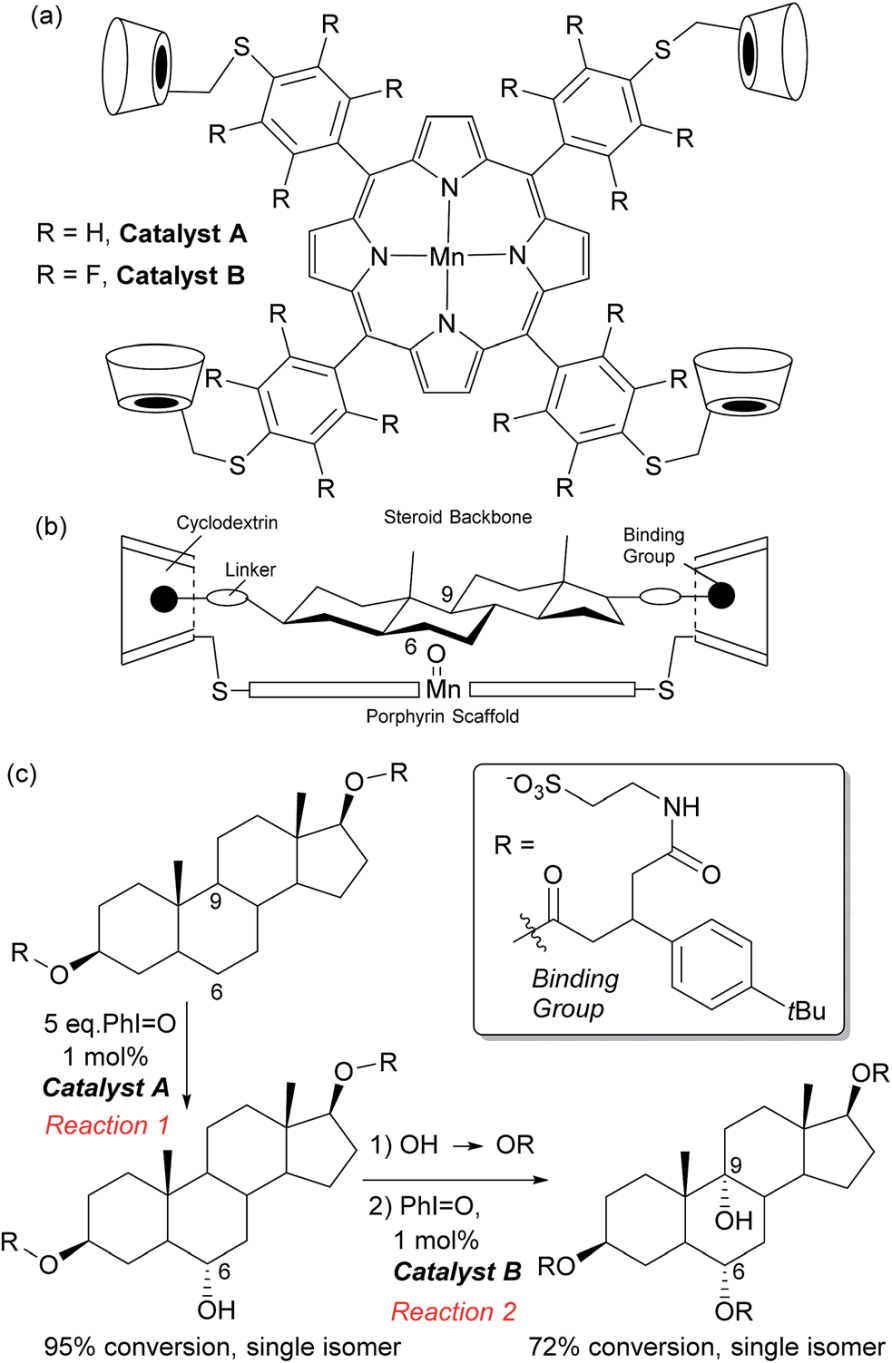
(a) Structure of manganese porphyrin catalysts developed for steroid functionalisation. (b) Outline of design principle used, involving binding groups on the steroid substrate to interact with cyclodextrin units on the porphyrin catalyst. (c) Selective oxidation at C-6 followed by installation of a further binding group then selective oxidation at C-9.

In Nature monooxygenases such as cytochrome P450 hydroxylase use a reactive metal centre to selectively oxidise a particular C–H bond in the presence of numerous others through precise positioning of the substrate *via* multiple non-covalent interactions at the enzyme active site.^61,62^ It has long been a goal of synthetic chemists to emulate such processes, but this presents significant challenges, not least the requirement for a strong oxidant to be used in concert with the delicate interactions required to function in an analogous manner to enzymes.^14,63^ The group of Breslow has carried out much pioneering work on emulating the action of enzymes using chemical systems, in particular towards site-selective functionalisations, some of which could be rendered catalytic.^13^ In 1997 they disclosed what they described as an artificial P-450 enzyme, in the form of a manganese porphyrin with four cyclodextrins attached (Fig. 10a).^64,65^ The cyclodextrins act as binding sites for a substrate suitably functionalised with 4-*tert*butylphenyl groups that interact *via* the hydrophobic effect. With these two interactions in place, they hypothesised that the reactive manganese centre is located directly over the steroid skeleton. If the binding geometry is well defined, site-selective and stereoselective C–H oxidation should result (Fig. 10b). They found that this is indeed the case and only the equatorial 6α secondary alcohol forms upon oxidation of the original test substrate, with perfect site- and stereoselectivity (Fig. 10c, reaction 1). Using modelling, they reasoned that selectivity arises due to presentation of the edge of the steroid skeleton to the reactive metal centre and that if the face could be presented instead, then this may result in oxidation of the C-9 axial hydrogen. Accordingly, they introduced a third binding interaction by functionalising the 6α-hydroxyl group introduced using their first oxidation. Oxidation of this compound using a more robust polyfluorinated porphyrin catalyst (catalyst B) then proceeded to quantitatively give the desired C-9 oxidised compound, a tertiary alcohol, again with exclusive site-selectivity (Fig. 10c, reaction 2).^66,67^ The same group has reported a number of other strategies for steroid functionalisation employing non-covalent interactions, although in many of these cases catalytic turnover was elusive.^68–70^

**Fig. 11:**
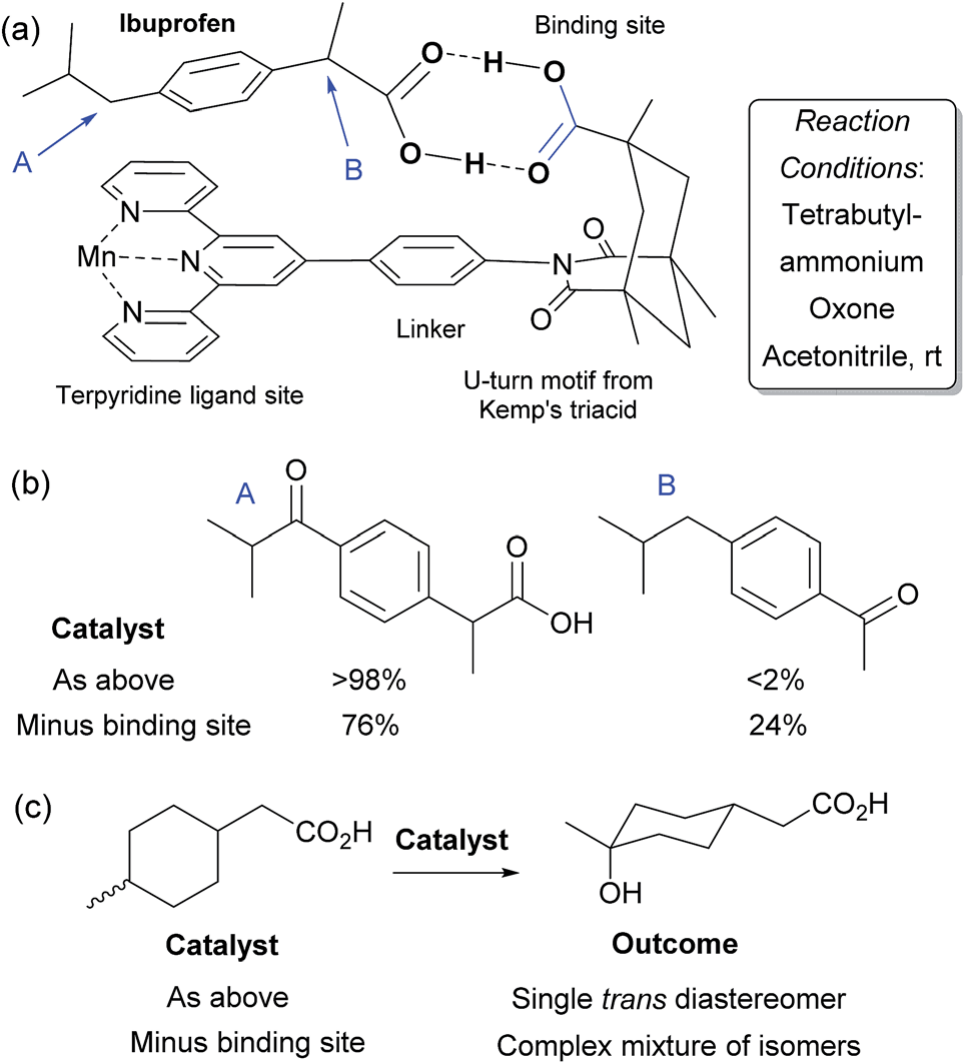
(a) Outline of catalyst structure (only one half of μ-oxo dimer shown for clarity) with components highlighted, in a hydrogen bonded complex with ibuprofen. (b) Results for oxidation of ibuprofen using catalyst depicted in (a) as well as a control variant without the binding site portion. (c) Outcome of oxidation of a mixture of *cis* and *trans* 4-methylcyclohexyl acetic acid using the same two catalysts.

In 2006, Crabtree, Brudvig and co-workers reported a selective C–H oxidation in which an intrinsic functional group in the substrate was used to interact with the catalyst scaffold, rather than requiring a preinstalled dedicated binding group.^71,72^ They aimed to selectively functionalise ibuprofen and designed a catalyst scaffold derived from Kemp's triacid combined with a terpyridine ligand site for manganese (Fig. 11a). On formation of a catalyst–substrate complex through dual hydrogen bonds, it was hypothesised that one particular benzylic position would be placed over the Mn centre and thus selectivity could be achieved through proximity. This was shown to be the case and >98% selectivity was obtained for oxidation of the position remote from the carboxylic acid group (Fig. 11b). A variant of the catalyst without the Kemp's triacid-derived binding site gave only 3 : 1 selectivity. They also examined their catalyst for oxidation of non-benzylic C–H bonds and found that on a mixture of *cis* and *trans* 4-methylcyclohexyl acetic acid, a single isomer was obtained with the optimal catalyst whilst a complex mixture results from the control variant which is unable to bind (Fig. 11c). These transformations have also been the subject of a detailed computational study in which it was shown that the double hydrogen bond perseveres throughout the whole reaction pathway and stabilises the entire potential energy surface.^73^

Although primarily pursuing enantioselectivity, Bach and co-workers reported a chiral C2-symmetric Rh(ii) complex containing remote lactam binding sites that was able to provide some site-selectivity in C–H amination.^74^ On a lactam-based substrate with two different benzylic positions, their catalyst was able to achieve 37 : 63 ratio of amination between the two whilst Rh_2_(esp)_2_ gave a 61 : 39 ratio.

### 3.2 Site-selective alkene functionalisation

As discussed in the previous section, mimicking the selective oxidising ability of cytochrome P450 enzymes has long been a goal of synthetic chemists. In this section, we will examine selective oxidation chemistry in the context of site selective alkene oxidation to give epoxides. Building on their previous studies in enantioselective photochemistry,^75^ Bach and co-workers have reported elegant studies towards enantioselective alkene epoxidation using a hydrogen bond-directed approach, which is also able to exhibit some control over site-selectivity in certain substrates containing two alkene groups.^76^

**Fig. 12:**
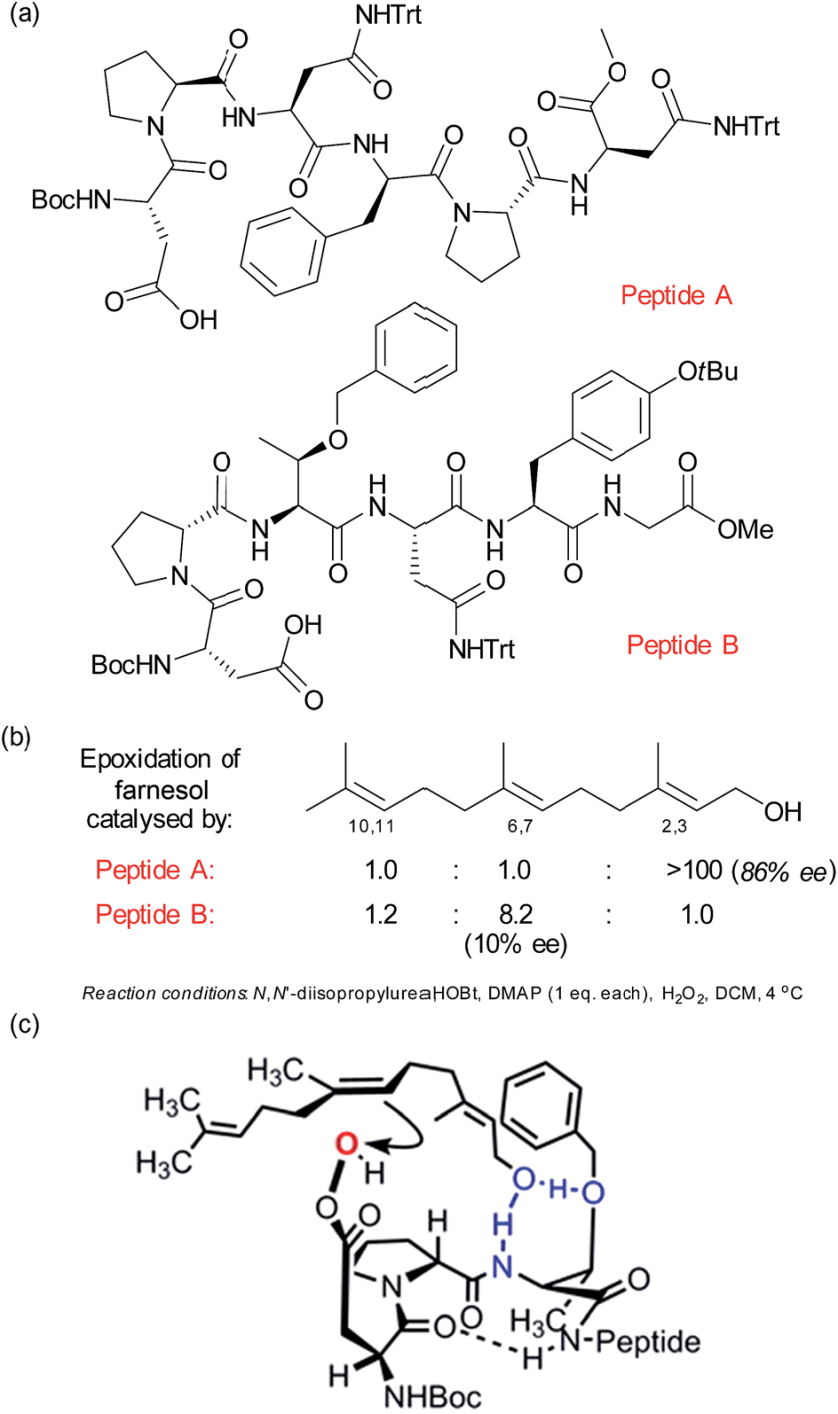
(a) Structures of two peptides identified to give complementary site-selectivity for epoxidation of farnesol. (b) Site-selectivity for farnesol epoxidation using peptides A and B. (c) One of several plausible models advanced to account for site-selective epoxidation of the 6,7 alkene of farnesol using peptide B. Reprinted with permission from ref. 78. Copyright 2014 American Chemical Society.

Related to the peptide catalysis discussed in Section 2.4 for regioselective ketone oxidation, the Miller group have develop a pair of peptide catalysts (Fig. 12a) that were able to distinguish between the 2,3 and 6,7 alkenes in the epoxidation of geranylgeraniol and farnesol (Fig. 12b).^77^ As discussed previously with respect to the peptide-catalysed Baeyer–Villiger oxidation, the epoxidation proceeds by the carboxylic side chain of a terminal residue being activated with a carbodiimide and then attacked by hydrogen peroxide to form a transient peroxyacid at the peptide terminus. The peptides were discovered through a combinatorial and on-resin screening approach which aimed to emulate the directed evolution of enzymes. The first two libraries culminated in peptide A, which was highly regioselective for the 2,3 alkene as well as having very impressive enantiomeric excess (Fig. 12b). Also of interest was a peptide which gave moderate selectivity for the 6,7 alkene and which when evaluated off-resin with a terminal methyl glycine unit (peptide B) gave a very impressive 8.2 : 1 ratio for the 6,7 over the 2,3 alkene. Interestingly, the latter epoxidation gave very low enantioselectivity, which was attributed to the high number of rotational bonds between the hydroxyl group and the alkene in this case, since the alkene is rather remote from the only functional handle in the molecule. A subsequent detailed analysis of the peptide libraries evaluated for this transformation, coupled with NMR analysis of the catalyst structure and evaluation of truncated analogues allowed the authors to propose several plausible models to account for the selectivity for epoxidation of the 6,7 alkene. One of these is depicted in Fig. 12c, in which the peptide possesses a β-turn conformation.^78^

### 3.3 Site-selective reactions of polyols

**Fig. 13:**
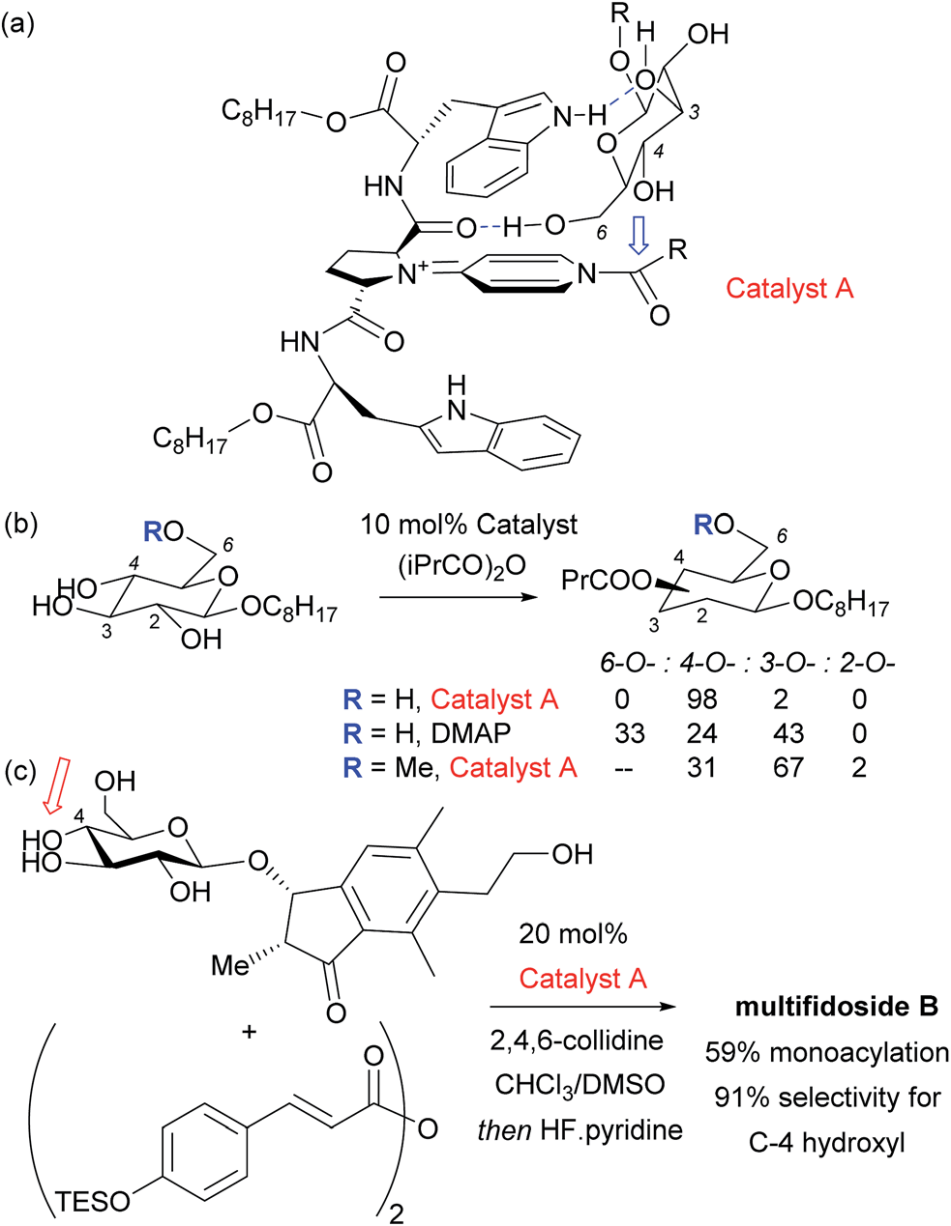
(a) Proposed model for highly site-selective C-4 acylation of glucopyranoside primarily *via* catalyst interaction with C-6 hydroxyl. (b) Details of site selectivity comparing catalyst A and DMAP as well as the effect of methylating the C-6 hydroxyl. (c) Total synthesis of multifidoside B through selective final step acylation in the presence of two primary hydroxyl groups.

Given the ubiquity of polyols in nature and medicine, most obviously in carbohydrate chemistry, the ability to selectivity functionalise one particular hydroxyl group in the presence of one or more others can be a very powerful tool. Indeed, transformations of carbohydrates are hampered by the often lengthy and elaborate protection/deprotection strategies that must often be followed to selectively modify hydroxyl groups.^79^ The same is true for elaboration of complex natural products for which the only practical way to access analogues is by modifying the structure as isolated from nature. Developing site-selective chemistry to address these challenges is a particularly difficult but potentially rewarding area and employing non-covalent interactions to do this is an obvious strategy since there must be, by definition, at least one other hydrogen bond donating group in the molecule which may act as an interaction point with a suitably designed catalyst. To first focus on the selective modification of carbohydrates, Kawabata and co-workers developed a multifunctional nucleophilic catalyst that possesses a nucleophilic pyridine but also hydrogen bond accepting amides which could potentially interact with hydroxyl groups in the substrate, to provide a directing effect.^80^ They rationalised that in glucopyranoside derivatives, the primary C-6 hydroxyl should be the most accessible and should preferentially form a hydrogen bond with a catalyst amide oxygen, thus holding the C-4 hydroxyl in close proximity to the electrophilic carbon of the catalyst *N*-acyl pyridinium ion (Fig. 13a). This proved highly effective and resulted in extremely high site selectivity for C-4 acylation, whilst DMAP gave mixtures of C-6, C-4 and C-3 monoacylation (Fig. 13b). In support of their mechanistic hypothesis, methylation of the C-6 hydroxyl resulted in poor C-3 *vs.* C-4 selectivity, similar to that provided by DMAP. The same group have recently used their catalyst to carry out several efficient total syntheses of natural glycosides, including the first total syntheses of multifidosides A–C in a very concise manner. The final step site-selective acylation occurs even in the presence of two primary hydroxyl groups (Fig. 13c).^81,82^

**Fig. 14:**
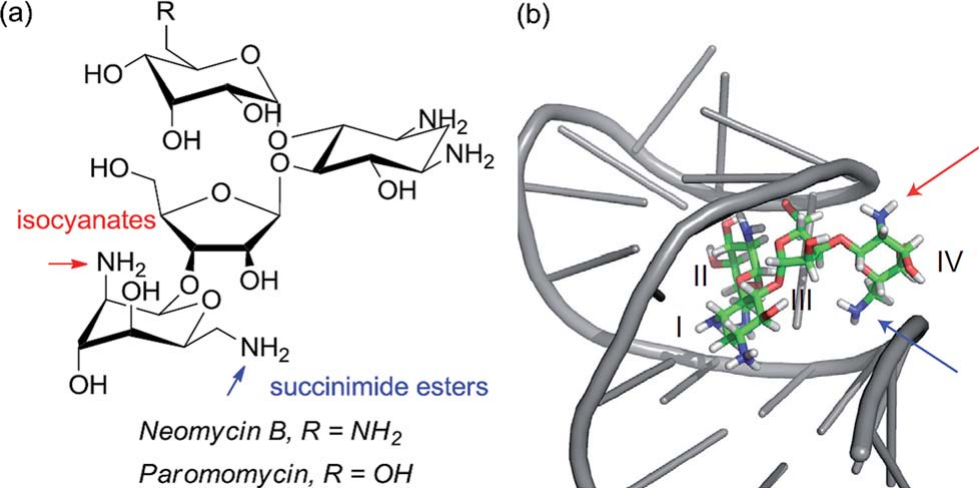
(a) Structure of aminoglycoside targets with blue and red arrows indicating sites of functionalisation after treatment with the aptamer and then either a succinimide ester (blue) or isocyanate (red). (b) Neomycin B bound to one of the RNA aptamers employed in the study, with the two accessible amines. Reprinted with permission from ref. 83. Copyright 2012 Nature Publishing Group.

Although not a catalytic reaction, a report by Herrmann and co-workers will be mentioned as it demonstrates a very elegant use of multiple non-covalent interactions to permit site selective acylation of several complex aminoglycoside antibiotics.^83^ Their approach uses what they refer to as an ‘aptameric protective group’ which comprises an RNA aptamer, an oligonucleotide that binds to a specific target molecule using non-covalent interactions. By using RNA aptamers established to bind to their specific aminoglycoside targets, they were able to sterically shield large portions of the molecule permitting selective acylation of the two amino groups in the lower portion of the molecule. Succinimide esters were found to selectively react with the primary amine whilst isocyanates went for the secondary amine (Fig. 14).

**Fig. 15:**
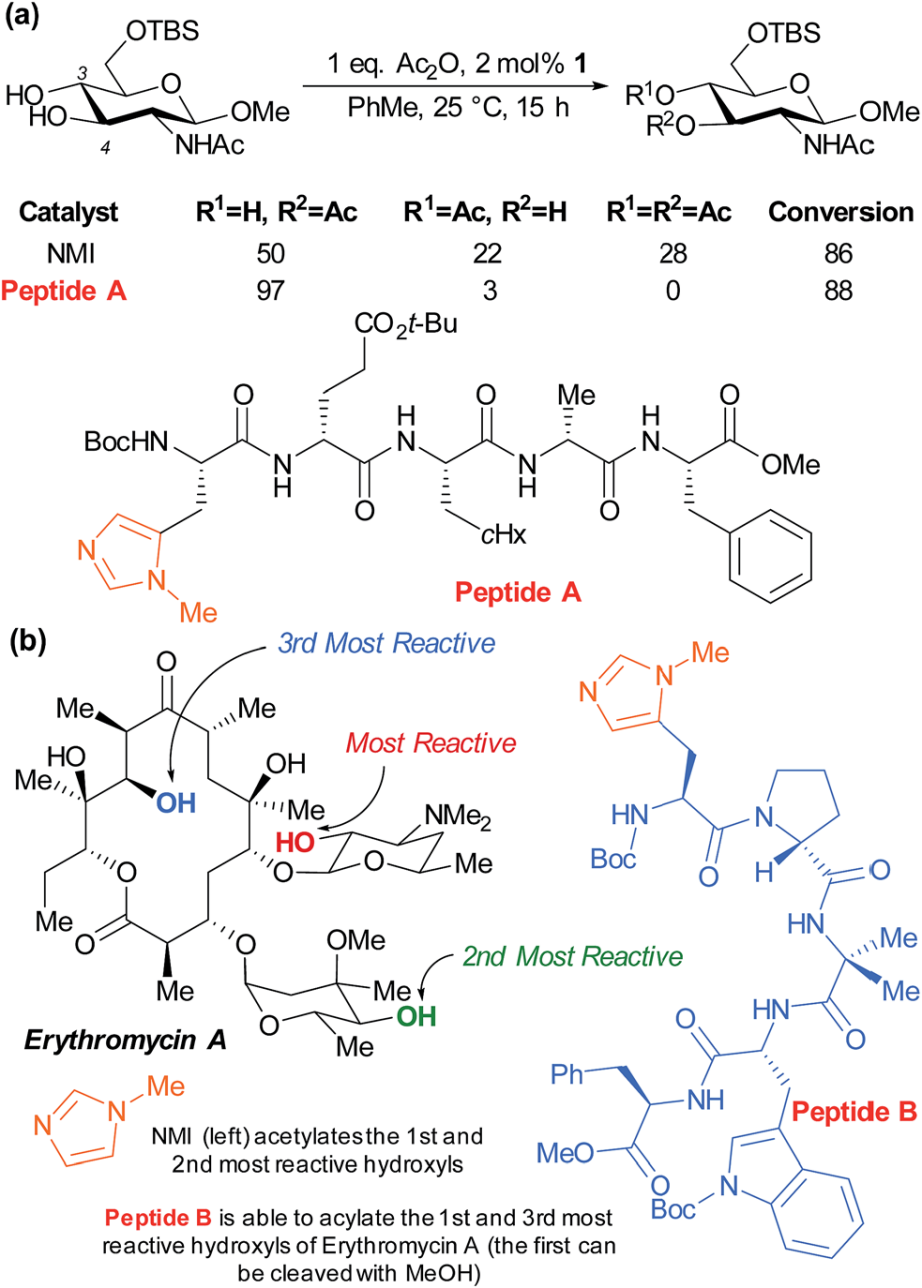
(a) Selective acetylation of the C-4 hydroxyl of a partially protected glucosamine using pentamer peptide A, a catalyst discovered though combinatorial screening. (b) Results of studies to differentiate the three secondary hydroxyls of antibiotic erythromycin A.

The Miller group have also very successfully applied their peptide catalysts to site selective derivatisations of polyols. In early studies, the acylation of the two competing hydroxyl groups of a partially protected glucosamine was investigated by screening against a peptide library with the aim of perturbing the product distribution *versus* the standard nucleophilic catalyst *N*-methylimidazole (NMI). Peptide A was observed to greatly enhance the selectivity for the marginally more reactive C-4 hydroxyl from ∼2 : 1 to >30 : 1, as well as inhibiting diacetylation (Fig. 15a).^84^ The same group subsequently sought to apply their peptide catalysts to the site selective acylation of significantly more complex polyol natural products and turned their attention to the antibiotic erythromycin A (Fig. 15b). Following initial reactivity studies using NMI, they found that with careful peptide selection, acylation of the third most reactive hydroxyl in the structure can be achieved using pentameric peptide B followed by a MeOH quench to autocatalytically cleave the first acetylation.^85^

**Fig. 16:**
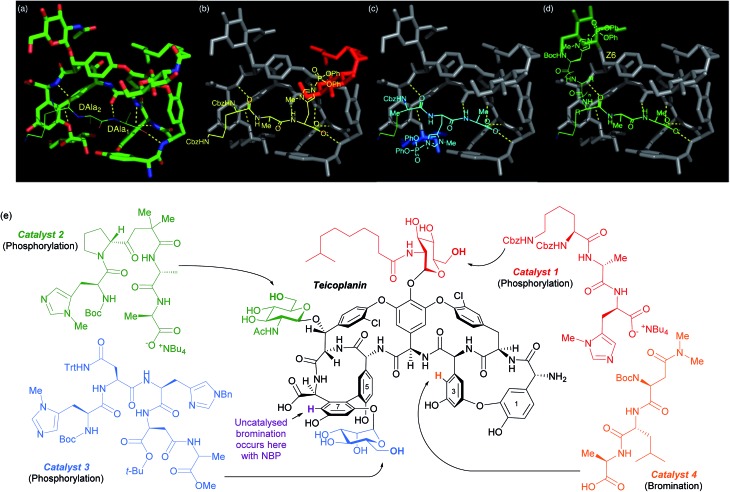
(a) Crystal structure of teicoplanin complexed with a DAla–DAla peptide. (b–d) Extrapolation of this binding mode to incorporate phosphoryl delivery from histidine in different positions in order to access each sugar, as initial hypothesis for rational design of peptide catalysts. (e) Selective phosphorylation of teicoplanin by catalysts 1, 2 and 3. Also shown are sites of bromination with NBP, both uncatalysed (purple) and using catalyst 4. Reprinted with permission from ref. 86. Copyright 2013 American Chemical Society.

The same group has shown that the structurally intricate antibiotic teicoplanin, which contains three remotely located glycopeptide units, can be selectively phosphorylated at each of the three primary hydroxyls independently, simply by switching between three peptide catalysts (Fig. 16e).^86^ The rational design hypothesis that preceded their study will be discussed as it provides a helpful visualisation as to how these peptides catalyst likely operate. In previous studies into the mechanism of action of this class of antibiotics, specific importance had been attached to an association between teicoplanin and a DAla–DAla moiety of the growing bacterial cell wall. By analysing X-ray crystallographic data of a DAla–DAla containing peptide bound to teicoplanin (Fig. 16a), the authors speculated that replacement of the DAla_1_ residue with histidine should put the nucleophilic imidazole in close proximity to the *N*-decanoylglucosamine ring (red, Fig. 16b) and doing the same for DAla_2_ may put the imidazole close to the mannose ring (blue, Fig. 16c). The long distance of the *N*-acetylglucosamine ring (green, Fig. 16d) away from the DAla–DAla binding site made the structure of a catalyst able to reach this ring more difficult to predict. The hypothesis led directly to catalyst 1, whilst catalyst 2 and catalyst 3 were ultimately discovered through a more conventional screening approach. The same group showed in a separate study on the same molecule that the uncatalysed site of bromination using *N*-bromophthalimide (NBP) on ring seven can be overridden by use of peptide catalyst 4 to favour ring three bromination (Fig. 16e).^87^ A similar approach has also been explored for the site selective thiocarbonylation^88^ and bromination^89^ of vancomycin.

## 4 Conclusions and outlook

The literature that has been summarised in the preceding sections highlights the progress that has been made thus far in using non-covalent interactions to address challenges of regiocontrol (Section 2) and site-selectivity (Section 3) in catalytic reactions.

In terms of regioselectivity, recent efforts in controlling C–H activation of arenes have focussed on iridium-catalysed borylation most likely due to its mild conditions, compatibility with non-polar reaction solvents and lack of requirement for acidic additives in the reaction protocol. These considerations make it ideal to explore in combination with potentially subtle directing effects such as hydrogen bonds or ion pairs. Given the numerous methods for arene C–H activation that have been developed in the last two decades, many using palladium, a key future challenge will be in applying these long-range directing concepts to C–H activation reactions that do not operate under such mild, neutral conditions. If this challenge can be addressed, then the potential for using long-reaching non-covalent interactions to direct C–H activation to arene *meta* and *para* positions is significant; most existing methods are based on cyclometalation and thus result in *ortho* functionalisation. As discussed in Section 2.3, examples of non-covalent regiocontrol in alkene functionalisation have focussed hydroformylation and these examples demonstrate how powerful a non-covalent directing approach can be in combination with careful catalyst design. Given the ubiquity of alkenes and the multitude of transition metal catalysed reactions that they undergo, it seems likely that there are many more opportunities to exert regiocontrol through suitably designed ligands that are able to undergo non-covalent interactions with the substrate.

Considering site-selective modification of C–H bonds, the initial advances made in the field of selective C–H oxidation by Breslow and co-workers are highly relevant to the challenges facing the field of C–H activation today.^64^ The difference at this point some two decades or more on is that there are substantially more methods for aliphatic C–H activation now developed and thus there exists great potential for combining these new methods with suitably designed ligands for non-covalent direction. Despite the remarkable advances made in metal-catalysed C–H activation, many still rely on cyclometalation approaches that result in proximal isomers. Reaching further to access distal products is arguably best achieved using non-covalent rather than covalent approaches, since building an elaborate long range directing group into a substrate significantly reduces practicality.^16^

The use of peptides as organic catalysts to permit site selective elaboration of polyols is perhaps the closest emulation of the way that natural enzymes work within the examples considered in this article. Their simple synthesis through amide bond formation permits investigation of large libraries which can be refined until the desired selectivity can be achieved. Whilst organic functionality at the ‘active site’ within the peptide has enabled acetylation, bromination and epoxidation reactions, very recent work from the Miller group has also demonstrated that a metal binding site can be incorporated into the peptide to enable directed transition metal catalysis across long distances.^90^ In this case the application was in enantioselective desymmetrisation but clearly demonstrates exciting potential for long range site-selectivity using an appropriately reactive transition metal bound to the peptide.

In closing, one could argue that an intrinsic disadvantage of the approach considered herein is that a functional group is required in the molecule to act as the ‘handle’ to interact with the catalyst through the non-covalent interaction. But a counter-argument would be that as long as the functionality is a common and useful functional group, then this should actually be seen as an advantage – after all the production of functionalised molecules is desirable. However, it should be borne in mind that a wider variety of catalysts may need to be developed to be able to cope with the substrate functionality being located in different positions, something that should be feasible with well rationalised and understood catalyst design.
